# Macrophages Orchestrate Hematopoietic Programs and Regulate HSC Function During Inflammatory Stress

**DOI:** 10.3389/fimmu.2020.01499

**Published:** 2020-07-23

**Authors:** Allison N. Seyfried, Jackson M. Maloney, Katherine C. MacNamara

**Affiliations:** Department of Immunology and Microbial Disease, Albany Medical College, Albany, NY, United States

**Keywords:** macrophage, hematopoiesis, HSC activation, niche, bone marrow, aplastic anemia, monocyte, inflammation

## Abstract

The bone marrow contains distinct cell types that work in coordination to generate blood and immune cells, and it is the primary residence of hematopoietic stem cells (HSCs) and more committed multipotent progenitors (MPPs). Even at homeostasis the bone marrow is a dynamic environment where billions of cells are generated daily to replenish short-lived immune cells and produce the blood factors and cells essential for hemostasis and oxygenation. In response to injury or infection, the marrow rapidly adapts to produce specific cell types that are in high demand revealing key insight to the inflammatory nature of “demand-adapted” hematopoiesis. Here we focus on the role that resident and monocyte-derived macrophages play in driving these hematopoietic programs and how macrophages impact HSCs and downstream MPPs. Macrophages are exquisite sensors of inflammation and possess the capacity to adapt to the environment, both promoting and restraining inflammation. Thus, macrophages hold great potential for manipulating hematopoietic output and as potential therapeutic targets in a variety of disease states where macrophage dysfunction contributes to or is necessary for disease. We highlight essential features of bone marrow macrophages and discuss open questions regarding macrophage function, their role in orchestrating demand-adapted hematopoiesis, and mechanisms whereby they regulate HSC function.

## Introduction

At homeostasis the bone marrow is a dynamic tissue housing stem and progenitor cells that give rise to components of the blood and the immune system via a measured and continuous process called hematopoiesis (1, 2). The bone marrow is a unique tissue; it is the origin for our immune cells, a site where billions of cells die and are removed, the production-site for red blood cells and platelets, and it is also the home for memory lymphocytes and plasma cells. Cell proliferation and differentiation, cell death and clearance, and cell migration create a dynamic microenvironment in the bone marrow. A variety of hematopoietic and non-hematopoietic cells interact in the bone marrow creating cellular niches that support hematopoietic stem cell (HSC) and progenitor cell (HSPC) survival and differentiation. HSCs and HSPCs reside at the apex of the hematopoietic hierarchy, giving rise to all cells of the blood and immune system. In healthy conditions, blood production relies on the contribution of HSPCs, whereas long-term HSCs remain quiescent, dividing rarely (3), and contributing little to steady-state blood production (4). Distinct populations of HSPCs can be characterized by expression of specific receptors and proteins, corresponding to lineage-biased blood production as observed in transplantation assays (5, 6). These findings support the notion that specific populations of HSCs/HSPCs are primed for producing certain cell types, though inherent plasticity exists in these populations. In response to trauma or injury, phenotypic changes and altered functional outputs demonstrate the flexibility of the hematopoietic compartment and its ability to be tailored to meet specific demands.

Inflammation, via production of inflammatory factors including cytokines and interferons, has been demonstrated to impact the differentiation and function of HSCs and HSPCs, highlighting the incredibly adaptive nature of the hematopoietic system. In direct response to pattern recognition receptor (PRR) stimulation (7, 8), cytokine signaling (9), and interferon stimulation (10–14), HSC and HSPC proliferative capacity, survival, and lineage commitment can change rapidly. An additional level of complexity exists *in vivo* as HSCs and HSPCs reside in particular regions or niches that provide signals to direct proliferation, differentiation, and/or quiescence. Here we focus on the unique role of macrophages in directing HSC and HSPC function and regulating hematopoietic responses during organismal stress, such as infection, autoimmunity, and aging. The ability to modulate HSC function by targeting the niche is important for clinical procedures, such as HSC mobilization, and holds potential for treating blood disorders including hematopoietic malignancies and marrow failure.

## Macrophages: Managers of Tissue Homeostasis

Macrophages are a heterogenous population of terminally differentiated cells that can be found in all tissues, including the bone marrow, where they perform essential homeostatic functions that include tissue remodeling, clearance of dead cells, and production of angiogenic factors (15). Macrophages are members of the mononuclear phagocyte system, which also consists of monocytes and dendritic cells (16). Named for their remarkable phagocytic capacity described over a century ago by Metchnikoff (17), macrophages express a variety of receptors that enable efficient phagocytosis, including complement receptors, Fc receptors (for immunoglobulins), and scavenger receptors [reviewed in (18)]. In addition to their ability to “eat,” macrophages sense endogenous and microbe-associated danger and damage signals via pattern recognition receptors (PRRs) such as Toll like receptors (TLRs), lectin sensing receptors, mannose binding receptors, and cytoplasmic NOD-like receptors. With the profound array of receptors to both ingest and sense their surroundings, they are positioned to act as highly sensitive detectors of environmental stress. In response to the substances ingested and the danger- and pathogen-associated molecular patterns (DAMPs and PAMPs, respectively) encountered, macrophages produce a variety of soluble and membrane-associated factors that can act in the immediate vicinity to modulate cell function and biological activities.

### Developmental Origins of Tissue Macrophages

Tissue macrophages are diverse in their ontogeny, arising from the embryonic yolk sac, fetal liver, or from post-natal bone marrow. Primitive macrophage progenitors arise from the yolk sac to seed the developing tissues, including brain and fetal liver, giving rise to microglia in the brain and pre-macrophages in the fetal liver (19, 20). Notably, yolk sac-derived progenitors have macrophage, but not monocyte, potential and do not require a monocyte intermediate in becoming microglia (20). Subsequently, yolk sac-derived erythro-myeloid progenitors (EMPs), which contain macrophage, as well as granulocyte/monocyte and mast cell potential, arrive in fetal liver, where they undergo differentiation into pre-macrophages and monocytes (19–21). Fetal-liver pre-macrophages differentiate into fetal monocytes, emigrate from the liver, and, upon entering tissues, rapidly initiate transcriptional programs due to microenvironmental cues which drive maturation and specification into tissue-resident macrophage populations. In splenic red pulp, heme-sensing drives proteasomal degradation of Bach1, thus de-repressing *Spic* expression, the master transcriptional regulator for red pulp macrophage specification (22). Liver-resident macrophages, Kupffer cells, require liver X receptor (LXR)-α activation and *Id3* transcription (23, 24). Whereas, most tissue macrophages require M-CSF for survival, GM-CSF secretion by lung epithelium drives alveolar macrophage specification via PPAR-γ (25, 26). Underscoring the heterogeneity of tissue macrophages, the kidney contains at least three macrophage subsets with distinct ontogenies and inflammatory profiles (27). The conditions necessary for specification into bone marrow-resident macrophages are currently unclear. Deletion of *Spic* or *Irf8* partially block bone marrow macrophage specification (22, 28), but a universal transcriptional program for the bone marrow remains elusive, perhaps due to the heterogeneity of macrophages in this tissue.

Although there are few to no immunophenotypic markers useful for distinguishing embryonic- from HSC- or monocyte-derived macrophages, lineage-tracing, and transgenic models have revealed valuable insights about macrophage ontogeny. In a seminal study, Hashimoto et al. (29) demonstrated that microglia, lung, red pulp, peritoneal, and bone marrow macrophage populations arose independently of adult HSCs and monocytes in a self-renewing manner (29, 30). More recently, *Ms4a3*, a monocyte lineage-specific gene expressed in granulocyte-monocyte progenitors (GMPs), was used to track post-natal seeding of tissues with monocyte-derived macrophages and identified tissue resident macrophage populations that were not replenished by monocytes (including Langerhans cells, Kupffer cells, and microglia) at steady state (30). The authors further demonstrate that bone marrow macrophages arise from *Ms4a3*^−^ monocyte-dendritic cell progenitors (MDPs), which did not share ontogeny with monocytes derived from *Ms4a3*^+^ GMPs, indicating that steady state tissue-resident macrophages and monocytes arise from separate lineages. These studies further demonstrated three modes of macrophage replacement in post-natal mice as they reach adulthood: fast replacement (blood, dermis, and gut), slow replacement (spleen, peritoneum, and kidney), or no replacement (microglia, epidermal Langerhans cells, and Kupffer cells) as predicted in previous studies (31, 32). The significance of the origin of distinct macrophage populations is a topic of intense interest as macrophages derived from embryonic hematopoiesis may be functionally distinct from those derived from adult hematopoiesis, and how distinct macrophage populations, monocyte-derived or tissue resident, contribute to health, and disease is unclear. In order to study the contribution of embryonic macrophages, macrophage egress was blocked from fetal liver by deleting the gene encoding plasmalemma vesicle-associated protein (PLVAP). PLVAP is the protein responsible for forming fenestrae in liver sinusoidal endothelium which allows pre-macrophages to migrate from the fetal liver to seed other tissues (33, 34). Adult mice lacking PLVAP display a striking absence of fetal liver-derived macrophage populations in the spleen, peritoneum, liver and lung, while tissue macrophages derived from adult hematopoiesis and yolk sac-derived microglia were unaffected. This study demonstrated that macrophage ontogeny can carry functional ramifications as pathologic levels of iron storage were observed in 5 week-old *Plvap*^−/−^ livers and spleens, despite unimpaired entry of HSC-derived monocytes. Thus, embryonic liver-derived tissue-resident macrophages may have a unique capacity for iron trafficking in adult tissues, and this mouse model may be particularly useful in examining bone marrow macrophage ontogeny.

When steady state conditions are interrupted, as seen in response to inflammation or injury, tissue resident macrophages of embryonic origin may be replenished by monocytes in a competitive manner. Partial depletion of embryonically derived Kupffer cells in the adult liver enabled monocytes to replenish the empty niche over several days and differentiate into cells largely resembling their embryonically-derived counterparts, differing only subtly in their expression of genes related to efferocytosis, pathogen recognition, and heme scavenging (35). The remaining embryonically-derived Kupffer cells became more proliferative and increased in number during this period (35), similar to observations in the heart (36), lung (37), and brain (38) following infection or injury. Thus, adult monocytes have the capacity to infiltrate most tissues and replace embryonically-derived macrophages. Questions remain as to whether replacement of bone marrow resident macrophages requires monocyte infiltration from circulation, or whether they mature *in situ* without ever leaving the bone marrow.

### Macrophage Functional Plasticity

Depending on the signals they receive, macrophages can adopt unique functional characteristics ranging from classical or pro-inflammatory activation (referred to as M1 polarization), to alternatively activated or pro-resolving/M2 phenotypes. The concept of “polarization” was proposed to describe *in vitro* differentiated macrophages and classify distinct functional and phenotypic programs (39), though these programs are vastly more complicated and nuanced *in vivo* (40, 41). Tissue-resident macrophages typically exhibit expression of *Arginase1* (42) consistent with their ability to proliferate in response to Th2 cytokines, such as IL-4 (43), and secrete IL-10 and TGFβ driving their ability to dampen inflammation and drive wound healing (40). Classically activated macrophages, induced by Th1 cytokines such as IFNγ and TLR4 stimulation by bacterial lipopolysaccharide (LPS), secrete IFNγ and tumor necrosis factor (TNF) and express *inducible nitric oxide synthase* (*Nos2*) thus enhancing the respiratory burst, and facilitating the killing of intracellular pathogens. The diversity of macrophage phenotypes observed *in vivo* blurs the current paradigm of polarization and highlights the context-dependence of macrophage function.

Clearance of apoptotic cells by macrophages (i.e., efferocytosis) is a fundamental process that accounts for the efficient and continuous removal of billions of dead, damaged, or unwanted cells each day. Efferocytosis is mediated by the recognition of apoptotic cells via exposure of phosphatidylserine (PtdSer) on the plasma membrane and through the release of factors that act as “find me” signals (44). Apoptotic cells can be sensed directly by phagocytes, via PtdSer receptors including the T cell immunoglobulin and mucin (TIM) family of receptors and others (45), or are recognized by phagocytes indirectly via receptors that bind protein S or Gas6, plasma proteins with affinity for PtdSer that act as bridging molecules [reviewed in (46)]. Stimulation of Tyro3, Axl, and MerTK (TAM) receptor signaling activates expression of suppressor of cytokine signaling (SOCS) proteins, inhibitors of signaling downstream of TLRs and interferon receptors (47, 48). In addition, MerTK signaling promotes the synthesis and release of pro-resolving lipid mediators by altering metabolic pathways (49, 50), and mice lacking MerTK and Axl have greater basal levels of pro-inflammatory factors, including IFNγ and TNF (51). MerTK signaling also promotes macrophage survival, proliferation, and migration (52–54). Expression of TAM receptors is under control of cytokine receptors, thus appropriate temporal regulation of efferocytosis and pro-resolving macrophage phenotypes should occur sequentially upon inflammatory signaling (47).

Macrophages are poised to participate in the rapid response to damage and danger as they are equipped with machinery to both activate inflammation and promote wound healing and tissue regeneration. Whereas, inflammatory responses are critical for host defense and immunity, the capacity to restore homeostasis is essential for maintaining tissue integrity and functionality. Therefore, macrophages are essential “bookends” to the cyclic and episodic inflammatory events encountered throughout life, and as such, their function may reveal insight to understanding how inflammatory stress impacts HSC function.

## Macrophage-Dependent Regulation of the Hematopoietic System

### Macrophages Instruct HSC Emergence

Macrophages, along with primitive erythrocytes and megakaryocyte progenitors are among the earliest differentiated cell types to emerge in the yolk sac, prior to the existence of HSCs (19, 55). During the earliest stages of life macrophages are essential in shaping the developing embryo, primarily through their ability to remove and clear dead cells; without this essential function, profound defects occur in the developing brain and lung (56). Development of the blood system occurs in several stages, with the final wave being when HSCs emerge (57–59). In the aorta-mesonephros-gonad (AGM) region, a transient window of endothelial-to-hematopoietic lineage specification occurs allowing emergence of HSCs (58), and specific inflammatory signals, including IFNγ, are required for this process (60). Using *Csf1r* reporter mice EGFP-positive macrophages appeared in the mesenchymal tissue of the aorta and in the lumen prior to HSC emergence (61), similar to findings in human tissue (62). These observations suggest that primitive macrophages migrate through the vasculature, localize to the site of HSC emergence, and are instructed to accumulate prior to emergence of HSCs. Macrophages were positioned such that they interacted with c-Kit^+^ progenitors entering the lumen, supporting the conclusion that they promote HSC emergence from the endothelium (63). Specific chemokines mediated CX_3_CR1-dependent recruitment of a unique macrophage population to the AGM that exhibited characteristics of both resident macrophages and inflammatory macrophages (63). The inflammatory signaling pathways required during development and HSC emergence are reactivated during stress and injury and suggest macrophages possess powerful regenerative capacity that may impact HSC function in adults.

### Bone Marrow Macrophages and HSC Niches

**Figure 1:**
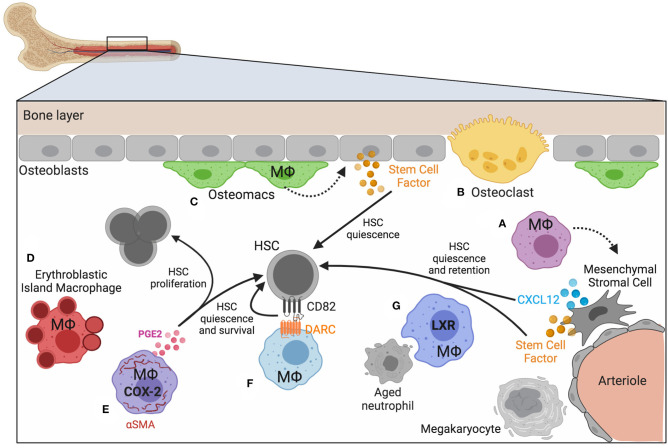
Macrophage heterogeneity in bone marrow and HSC niches. Homeostasis of blood production depends on bone marrow niches that regulate the location and function of HSCs and progenitors. Macrophages play both direct and indirect roles in maintaining HSC quiescence and maintenance. **(A)** Mesenchymal stromal cells respond to signals provided by nearby macrophages. **(B)** Specialized, multinucleated macrophages (osteoclasts, yellow) and **(C)** a distinct subset of macrophages have been identified near the bone lining (osteomacs; green). **(D)** Macrophages associate with developing erythroid progenitors (erythroblastic island macrophages; red), and **(E–G)** macrophages that express αSMA (purple), DARC (light blue), and LXR (dark blue) have been demonstrated to impact hematopoiesis and HSC function. Figure generated using BioRender.com.

The bone marrow contains defined anatomical niches that maintain HSCs via a complex network of secreted factors, cell-matrix contacts, and cell-cell interactions (64). These niches maintain HSCs through extracellular matrix and stem cell factor (SCF) secreted by osteoblasts, mesenchymal stromal cells (MSCs), and arteriolar endothelial cells (65–67). Other factors, such as CXCL12 promote HSC retention and quiescence (68). Macrophages are integrated throughout the bone marrow and exist in the various HSC niches that have been described. Macrophage crosstalk with Nestin^+^ niche cells results in *Cxcl12* transcription and, following macrophage depletion, expression of *Cxcl12* (as well as other HSC maintenance and retention factors *Angpt1, Kitl*, and *Vcam1*), is lost, and HSCs egress from the marrow (69). How macrophages modulate HSC niches is not yet fully understood, but as their function can impact the cell-cell interactions and the microenvironment, we suggest they play underappreciated roles in modulating HSC function. A variety of different macrophage populations have been described by both phenotype and function [reviewed in (70)] and their location and function in the bone marrow encompass a spectrum of biological roles with implications on HSC function (Figure 1).

### Macrophages in Endosteal Niches

Macrophages are key regulators of bone homeostasis, a dynamic process involving many cell types and continuous remodeling [reviewed in (64)]. The main bone forming cells, osteoblasts, derive from mesenchymal progenitors aided by macrophage signals (Figure 1A) and were among the first cell types demonstrated to regulate the size of the HSC pool (65). Osteoclasts are multinucleated bone resorbing cells that arise from the common myeloid progenitor lineage in response to M-CSF and RANKL signaling, and have recently been shown to be long-lived cells fusing with circulating monocytes [(71); Figure 1B]. Osteoclast-committed progenitor cells respond to the cytokine osteoprotegerin, which induces commitment to the osteoclast lineage (72). Whereas M2 polarizing signals promoted osteoblast differentiation and bone mineralization *in vitro*, M1-polarized macrophages had the opposite effect, thus demonstrating how macrophage-mediated inflammation impacts bone formation (73).

Distinct from osteoclasts are a subset of macrophages termed “osteomacs” that reside along the bone lining in close association with osteoblasts and megakaryocytes and were found to play key regulatory roles in modulating osteoblast function [(74); Figure 1C]. The interaction between macrophages and osteoblasts was necessary for low-level activation of nuclear factor κB (NF-κB) in osteoblasts and their ability to maintain HSCs through appropriate chemokine signaling (75). Osteomacs were found to be supported by the presence of megakaryocytes and their interactions together synergized with osteoblasts to regulate HSC repopulating potential as tested in transplantation assays (76). The supportive role that macrophages play in regulating a variety of biological processes in bone turnover and MSC function suggests that their activation state is an important regulator of HSC function.

### Erythroblastic Island Macrophages

Macrophages play a fundamental role in the development and life of red blood cells, supporting both the birth of erythrocytes and controlling their removal upon completion of their ~90-day lifespan (77, 78). Erythroblastic island (EBI) macrophages are a unique, EpoR-expressing population in the bone marrow [(79); Figure 1D]. These macrophages “nurse” committed erythroid progenitors (EPs) through the process of becoming enucleate reticulocytes via the provision of growth factors, potentially supplying iron that EPs incorporate into heme, and finally by engulfing and destroying the pyrenocyte, the product of asymmetric erythroblast cell division containing the nucleus, plasma membrane, and a small amount of hemoglobin-rich cytoplasm (80, 81). Among the many signals required for efficient erythropoiesis is EpoR (erythropoietin receptor) signaling, essential for the terminal maturation of erythroid-lineage cells in adult erythropoiesis (82). In addition to its expression on erythroid lineage cells EpoR is expressed by a variety of macrophage populations throughout the body, where its signals induce pleiotropic cytoprotective (83) and immunomodulatory effects (84). EpoR signaling in peritoneal macrophages enhanced PPARγ-mediated expression of efferocytosis machinery, increased TGFβ production, reduced production of IL-6, TNF, IFNα, and IFNβ, but did not significantly affect levels of IL-10, IL-12, or IFNγ (85, 86), reflecting specificity in macrophage signaling output. Moreover, imbalances in these cytokines were detected in the serum of adult mice in which macrophages lacked EpoR, which coincided with the development of a systemic, lupus-like disease (87).

EBI macrophages have been described as EpoR^+^ but if, and how, EpoR signaling in EBI macrophage functions relative to blood production is unknown. Relative to other BM macrophages, EpoR^+^ EBI macrophages were found to express elevated *MerTK* and *Ax*l (but not *Tyro3*) at steady state (79). Because EBI macrophages constantly efferocytose pyrenocytes, disrupted TAM receptor signaling in these cells during inflammatory stress may contribute to inflammatory anemias. A role for direct Epo signaling in HSPCs is not likely as mice expressing a constitutively active EpoR mutant, R129C, exhibited an expanded erythroid- and granulocyte/monocyte-lineage compartment, whereas other hematopoietic lineages were normal (88–90). Committed erythroid progenitors were recently found to localize near sinusoidal endothelium, occupying niches with HSCs and requiring signals from Leptin receptor^+^ MSCs (91). As macrophages likely share these niches with progenitors, their function and activation may contribute to both erythroid progenitor and HSC location. Consistent with this notion is the observation that macrophage depletion results in HSC mobilization (discussed in more detail below) as well as a profound loss in medullary erythropoiesis that coincides with extramedullary erythropoiesis. As macrophage depletion diminishes *Cxcl12* expression, it is possible that immediate release of HSCs into circulation occurs very rapidly from these erythroid and HSC niches.

### Direct Interactions With HSCs

A rare subset of macrophages expressing the fibroblastic marker, alpha-smooth muscle actin (αSMA), was shown to directly interact with primitive HSPCs in bone marrow [(92); Figure 1E]. Using an αSMA reporter mouse to identify vasculature niche pericytes, these macrophages were found to be migratory and more abundant upon stimulation with LPS. Furthermore, they expressed high levels of cyclooxygenase-2 (COX-2), which was important for preserving primitive HSPCs under inflammatory stress conditions, via PGE-2-dependent HSPC survival (93). One day post-radiation COX-2 expression and PGE-2 production increased among αSMA^+^ macrophages, and these changes were found to preserve HSC quiescence. The timing of this response was critical, however, as addition of PGE-2 at later stages was found not to be protective. PGE-2 can impart both pro-inflammatory and anti-inflammatory signals, depending on engagement with specific receptors, and also signals in many different cell types *in vivo*. Therefore, the impact of PGE-2 on HSCs *in vivo* is likely highly dependent on both the timing and cellular context.

Macrophage-dependent regulation of HSC quiescence may also occur via the tetraspanin CD82, expressed on HSCs, and its interaction with the pseudo-chemokine receptor Duffy-antigen associated receptor (DARC), expressed on a subset of macrophages in the bone marrow [(94); Figure 1F]. Tetraspanins have previously been shown to promote quiescence of HSCs (95) and CD82 was found to regulate HSC adhesion via altering integrin stability and density on the plasma membrane and ultimately enforcing dormancy that was dependent on TGFβ. Due to the lack of a G-protein coupling motif, DARC acts as a chemokine “sink” because it can bind chemokines but does not transmit signals (96), and as such may be primarily expressed by macrophages that are programmed to be anti-inflammatory, thus creating an anti-inflammatory microenvironment. DARC is expressed by endothelium and also found on red blood cells, and how DARC may regulate the local microenvironment in the bone marrow is a topic that needs further investigation.

In line with evidence that macrophages regulate HSC function *in vivo*, macrophage polarization was shown to impact HSC self-renewal *in vitro* (97). Using an *ex vivo* HSC expansion model, macrophages cultured with IFNγ or IL-4 were subsequently co-cultured with LSK cells to analyze cobblestone area-forming cells (CAFCs) and subjected to transplantation to evaluate function. Macrophages cultured with IFNγ increased expansion of LSK cells, but significantly reduced the number of CAFCs. In contrast, macrophages cultured with IL-4 reduced expansion of LSK cells but increased the number of CAFCs. Using *Nos2* and *Arg1* knockout mice the effects on LSK expansion and CAFC formation were reversed suggesting that increased HSC self-renewal is partly due to increased *Arg1* expression. HSC self-renewal was suggested to be due to an increase in spermidine, a result of arginase metabolism, and enhanced expression of *Ezh2* and *Gif1*, key genes related to self-renewal. Thus, the metabolic programing of macrophages can instruct HSC proliferation and determine self-renewing vs. differentiating cell division choices, and the precise molecular programming driving these fate choices will be important to decipher. As macrophages are integrated throughout the bone marrow niche and can interact directly with HSCs, defining the proximity and function of distinct macrophage populations may yield *in vivo* approaches to target HSC function.

## HSCs on the Move: Macrophages Regulate HSC Location

### HSC Mobilization

In healthy conditions HSCs and HSPCs are enriched in the bone marrow, though they are released at low numbers into circulation and can enter extramedullary tissues (98). This homeostatic mobilization is dependent upon circadian rhythm-dependent responses (99) and linked to the mobilization and clearance of neutrophils in the bone marrow (100, 101). The relatively short lifespan of a typical neutrophil requires not only rapid production but efficient removal of expired, or “aged,” neutrophils. Tracking the kinetics of neutrophils in the blood revealed dynamic modulation of newly released neutrophils and loss of phenotypic aged neutrophils that corresponded to circadian fluctuations (101). Clearance of neutrophils in the bone marrow induced changes in the local niche microenvironment, including reduced expression of CXCL12, and concurrent increase in blood progenitors. Macrophages were necessary for neutrophil clearance and activated LXR-dependent programs that promote apoptotic cell clearance (Figure 1G). This seminal study demonstrated a direct link between macrophage function and the homeostatic, daily release of hematopoietic progenitors. Lifestyle stress that interferes with normal circadian-dependent processes is, therefore, likely to impact HSC and HSPC mobilization, the consequences of which are not yet fully understood. It is intriguing to speculate how perturbation of macrophage phagocytic capacity, such as that observed during inflammation or resolution, impacts HSC function via regulation of the bone marrow niche.

Mobilization of HSCs and HSPCs can be rapidly induced by inflammatory signaling, a process that synchronizes with granulocyte production and their release into circulation (102–104). HSC mobilization and granulopoiesis can be induced by the growth factor granulocyte colony stimulating factor (G-CSF) that results in profound decrease in *Cxcl12* (105–107). G-CSF receptor signaling is not required in hematopoietic progenitors themselves (108), however, and G-CSF-driven HSC mobilization was found to require macrophages (109). G-CSF results in specific and profound decrease in bone marrow macrophages, whereas other tissues such as spleen do not experience this decrease (110, 111). Therefore, local loss of macrophages in the bone marrow promotes HSC mobilization, consistent with observations that clodronate-loaded liposome (Clod-lip)-mediated macrophage depletion induces HSC mobilization, and significantly enhanced G-CSF driven HSC mobilization (69). The increase in circulating HSCs in these conditions has been attributed to a reduction in the chemokine CXCL12 and other HSC retention signals (69, 112). The loss of these retention factors, some of which also enforce quiescence, may contribute to the transient expansion of the HSC/HSPC pool as Clod-lip-mediated depletion of macrophages was also found to correlate with increased HSCs/HSPCs in the bone marrow (113). Analysis of HSC proliferation and quiescence upon Clod-lip treatment revealed a transient increase in proliferation among phenotypic HSCs, followed by an increased proportion of quiescent HSCs, suggestive of self-renewing proliferation (113).

### Returning Home: Engraftment

In addition to regulating HSC mobilization, macrophages were found to be critical for bone marrow engraftment of HSCs following transplantation (114). A proportion of macrophages in bone marrow exhibited radio-resistance and relative persistence for more than 1 month post-radiation. These radio-resistant macrophages were necessary for efficient HSC engraftment and reconstitution of the hematopoietic system after lethal irradiation, as diphtheria toxin receptor (DTR)-dependent depletion of CD169-expressing macrophages reduced HSC engraftment. These studies highlight that bone marrow-resident macrophages can persist after lethal radiation. This finding impacts conclusions from studies where radio-resistant cells have been implicated in specific responses. It is not surprising that macrophages can persist subsequent to radiation as tissue-resident macrophages may be slowly cycling or not proliferating, and also have the capacity to self-renew in response to M-CSF. Expansion of host macrophages after radiation can be increased by exogenous M-CSF and reduces graft vs. host disease supporting a protective role for resident macrophages in reducing inflammation in the context of transplantation (115).

In a separate study, macrophage depletion was found to promote HSC recruitment and retention in the bone marrow leading to stable hematopoietic chimerism (116). While seemingly in contrast to the observation by Kaur et al. that macrophage depletion reduces HSC engraftment (114), the exposure to macrophage depletion in combination with lethal, ionizing radiation, likely induced a distinct pro-inflammatory microenvironment impairing engraftment. In contrast, Clod-lip-dependent macrophage depletion alone may not elicit the same extent of inflammation. These differences raise important caveats pertaining to interpretations of studies using macrophage depletion methods, as these techniques modulate inflammation and impact other cells *in vivo*. Macrophage depletion using Clod-lip has been studied by many laboratories and the mechanism of depletion involves apoptotic cell death of the liposome-engulfing cell upon intracellular release of the bisphosphonate (117). It is important to consider that the process of macrophage depletion via apoptosis will simultaneously generate many apoptotic cells *and* remove professional phagocytes that typically clear apoptotic cells. Increased apoptotic cells will lead to an accumulation of factors that act as “find me” signals priming surrounding cells for apoptotic cell clearance. It is also important to note that depletion models are not effective at ablating cell populations entirely and the homeostatic mechanisms maintaining tissue macrophages are immediately activated upon their depletion. It can be speculated that these changes alter phagocytic and efferocytic function of neighboring cells, contributing to an altered inflammatory state and, as such, may dampen inflammation. At the same time, CD169 expression, while more restricted to macrophage-lineage cells, does not appear to be limited to specific macrophage populations, is heterogenous among erythroblastic island macrophages (118), and has been found to change under inflammatory conditions (119). Thus, additional tools for targeting and manipulating macrophages *in vivo*, are required and there is a critical need to identify specific transcriptional networks for bone marrow macrophages.

### Macrophages and HSC Relocation

Mobilized HSCs may not return to the bone marrow and have the potential to migrate to and reside in other tissues. Extramedullary hematopoiesis occurs under physiological conditions, as seen during embryogenesis (24), and in adult tissues, such as the spleen and lung, where pulmonary thrombopoiesis has been observed (120). Extramedullary hematopoiesis may also arise secondary to various pathologic circumstances, such as bone marrow myelofibrosis, cancer, and myocardial infarction. Splenic red pulp macrophages sequester mobilized HSCs in the spleen via the adhesion molecule VCAM-1 following myocardial infarct (121), and these HSCs are monocyte-biased and express the chemokine receptor CCR2. An HSC-intrinsic effect may also contribute to HSC mobilization in this condition as ~15% of HSCs in bone marrow express the chemokine receptor CCR2 and over 90% of those found in blood and spleen are CCR2^+^ suggesting that bone marrow contains a subset of HSCs primed for mobilization via CCR2. As CCR2^+^ HSCs are myeloid biased (122), these data suggest that macrophages in the spleen support a reservoir of HSCs for rapid production and mobilization of myeloid cells, and this may be relevant to a variety of disease conditions.

## Macrophages Control Demand-Adapted Hematopoiesis

Organisms encounter many different types of stress in a lifetime, but one of the most consistent pressures is the exposure to microbes, many of which are pathogenic. The systemic response to injury and infection is exemplified by demand-adapted hematopoiesis, a process of expedited myeloid cell production (123, 124). This process of “emergency myelopoiesis” is a response to inflammation elicited either experimentally with sterile PRR agonists or via infection with pathogenic organisms, where myeloid progenitors rapidly proliferate, generating and mobilizing myeloid cells (125). Pro-inflammatory factors including TNF and IL-1 are critical for remodeling the bone marrow, such that resources support myeloid cell production at the expense of lymphopoiesis (123, 126). G-CSF signaling results in increased reactive oxygen species promoting emergency granulopoiesis (127). G-CSF drives a profound decrease in medullary macrophages that coincides with an expansion of the granulocytic lineage and their subsequent release, consistent with the observation that neutrophils and HSCs share CXCL12-dependent retention mechanisms (128). The role of non-hematopoietic cells in driving emergency myelopoiesis was illustrated by elegant experiments wherein IL-1R responsiveness was restricted to radio-resistant host cells (129). Additional studies determined that TLR4-driven emergency granulopoiesis involved endothelial cells and their ability to respond to LPS (130). As macrophages likely contribute to radio-resistant cells in mouse chimeric studies, it remains to be determined whether macrophages also respond to TLRs and IL-1 in the context of “emergency granulopoiesis.”

In response to intracellular pathogens that elicit type II interferon (IFNγ) responses, the acute impact of G-CSF-dependent macrophage depletion does not occur, and rather IFNγ maintains bone marrow macrophages and drives expedited production of monocytes (131–134). The ability to fine tune myeloid cell production may have evolved to combat different types of pathogens; for example, whereas neutrophils are critical for control of extracellular pathogens, monocyte-derived macrophages orchestrate cell-mediated immunity necessary for clearance of intracellular organisms. In the context of acute viral infection, IFNγ is necessary to restrict granulopoiesis and prevents viral encephalitis (135). These data suggest that IFNγ counters G-CSF-induced depletion of macrophages and would limit HSC/HSPC mobilization. Consistent with this idea, during infection with an intracellular bacterial pathogen that elicits a robust IFNγ response, the bone marrow, splenic, and blood pools of HSC/HSPCs are reduced, whereas similar infection in the absence of IFNγ or in mice where macrophages cannot respond to IFNγ robust HSPC mobilization is observed (113). Additional factors that contribute to the macrophage-mediated myeloid responses will be important to delineate to further our understanding of how demand-adapted hematopoiesis is controlled, and how these responses contribute to host defense and pathogenesis.

Platelets are essential for hemostasis, and these small granulocytes also play vital roles in immunity and inflammatory responses. Platelets are in high demand during acute infection and injury, and low platelet counts, or thrombocytopenia, is a risk factor associated with severe disease in sepsis (136–138). Platelets have the ability to directly trap pathogens (139, 140), and control early inflammatory responses to pathogens by regulating the migration, activity, and function of myeloid cells (141, 142). The host's ability to rapidly generate platelets in the context of inflammation is likely important for survival. Supporting the crucial role of platelets in immunity are the observations that inflammation can drive expansion of megakaryocyte-primed CD41^hi^ stem-like hematopoietic progenitors (143), enhance chemokine-mediated platelet production (144), and promote an altered form of IL-1 receptor-dependent platelet generation via the rupture of megakaryocytes (145). The observation that HSCs with inherent megakaryocyte bias exist at the top of the hematopoietic hierarchy supports the idea that platelets play an essential role in host fitness (146). IL-1 is a highly conserved component of innate immunity and IL-1α is essential for timely wound healing responses, in part via its ability to increase platelet production (147). Platelets can modulate macrophage function in a variety of settings, and can promote macrophage activation and clearance of bacteria (148–150). At the same time, macrophages can produce a variety of cytokines and chemokines that may act locally as a source of pro-inflammatory factors that influence platelet activity and possibly augment platelet production. Inflammatory conditions can induce expression of podoplanin on macrophages, which was shown to activate platelets in mice via the receptor CLEC-2 (151). Megakaryocytes also express CLEC-2 and signaling via this receptor promotes normal levels of thrombopoietin necessary for HSC maintenance (152). Megakaryocytes are not only the source of platelets but also direct regulators of HSC function and enforce HSC quiescence through production of CXCL4 and TGFβ (153, 154). As macrophages and megakaryocytes likely share common niches in the marrow it will be important to further understand their interactions, both contact-dependent and independent, and how these interactions impact megakaryopoiesis, thrombopoiesis, and HSC function.

## Macrophages in the Pathogenesis of Bone Marrow Failure Syndromes

**Figure 2:**
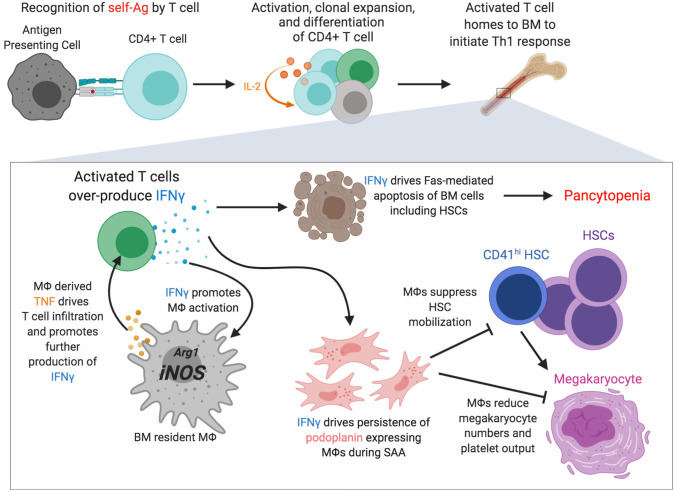
Macrophages are novel regulators of severe aplastic anemia pathogenesis. Recognition of self-antigens by particular T cell clones is thought to initiate severe aplastic anemia (SAA). Activation, clonal expansion mediated by an IL-2 feedback loop, and differentiation of the self-reactive T cell occurs and homing to and accumulation in bone marrow drives a strong Th1 response including overproduction of IFNγ, an essential driver of disease. IFNγ promotes Fas-mediated apoptosis of various bone marrow cells. IFNγ also promotes macrophage persistence and activation with increased *Nos2* transcription and increased TNF production. IFNγ drives the persistence of macrophages that express podoplanin which corresponds with decreased marrow megakaryocytes, reduced numbers of CD41^hi^ HSCs, and severe thrombocytopenia. Figure generated using BioRender.com.

Severe aplastic anemia (SAA) is a rare, auto-reactive T cell-driven bone marrow failure disease resulting in pancytopenia and accompanied by the loss of HSCs (155–157). Although the direct cause remains unknown, exposure to radiation, chemicals such as benzene and certain pesticides, and infections have been linked to SAA (158, 159). Due to numerous clinical overlaps with other blood diseases, SAA diagnosis is often complicated and accomplished via a process of elimination. Inflammation in the bone marrow during SAA is driven by both IFNγ and TNF (157, 160, 161). T cells are important drivers of disease via over-production of IFNγ, and T cell, derived IFNγ promotes Fas-mediated apoptosis of bone marrow cells contributing to bone marrow hypocellularity and loss of HSCs and HSPCs (160, 162, 163). Recent evidence has identified a role for macrophages in SAA pathogenesis [(119, 164); Figure 2]. In a murine model of SAA bone marrow resident macrophages were found to persist during disease despite loss of other myeloid cells (119), consistent with findings from human SAA patients (165). Macrophage persistence was dependent on IFNγR and M-CSF-R signaling, and disease was associated with emergence of a population of macrophages that express podoplanin (119). Macrophage persistence and dysfunction during SAA correlated with elevated *Nos2* expression and a loss of niche cells. Blocking IFNγ signaling in macrophages, or depleting macrophages, resulted in a striking but transient increase in platelet-biased CD41^hi^ HSCs that corresponded to markedly improved thrombocytopenia and rescued mouse survival. IFNγ is known to drive macrophage activation, promoting a pro-inflammatory phenotype and expression of TNF (40, 166). Macrophage-derived TNF was critical for donor T cell infiltration into the bone marrow and T cell secretion of IFNγ, and TNF-producing macrophages were found to be more abundant in SAA patients compared to healthy controls (164). Moreover, macrophages from SAA patients elicited more robust IFNγ responses by co-cultured T cells. The underlying molecular mechanisms whereby IFNγ-activated macrophages reduce HSCs may contribute to additional, more targeted therapies to treat SAA. IFNγ signaling in macrophages was necessary for increased production of pro-inflammatory beta-chemokines in the bone marrow during SAA (167) and how these chemokine gradients impact HSC location and function will be important to pursue to further understand SAA pathogenesis.

Myelodysplastic syndromes (MDS) are a heterogeneous collection of bone marrow failure diseases characterized by cytopenias, marrow dysplasia, and the expansion of mutation-carrying clones (168, 169). MDS is more common in older patients, correlating with the age-related development of clonal hematopoiesis, and much like SAA, marrow failure in MDS is characterized by a unique inflammatory signature including increased TNF, IFNγ, and cell death in the bone marrow (170, 171). Moreover, inflammasome activation was found to drive MDS demonstrating that cell death mechanisms, specifically pyroptotic cell death, contribute to MDS pathophysiology (172). Consistent with the complex regulation of hematopoiesis by both intrinsic mechanisms and niche-driven mechanisms, the bone marrow microenvironment plays a central role in the development of MDS (173–175). Monocytes from MDS patients were shown to generate macrophages that exhibit increased TNF production and altered phagocytic capacity (176), and dysfunctional macrophages are seen early in MDS and may be an initiating event that allows progression of MDS and leukemia.

Intrinsic drivers of MDS are suggested by the outgrowth of particular clones and numerous mutations have been described and linked with MDS prognosis (177). Ten eleven translocation 2 (TET2) is an epigenetic regulator frequently mutated in clonal hematopoiesis and MDS. *Tet2* is the most highly expressed TET enzyme in murine and human macrophages and loss of TET2 results in enhanced *Il1b* and *Il6* expression by macrophages in response to LPS stimulation demonstrating its role in controlling inflammation (178). Mutations in *Tet2* may alter inflammatory responses in the bone marrow microenvironment thus contributing to HSC dysfunction and clonal hematopoiesis leading to MDS. Tet2-deficient macrophages also exhibit elevated *Arg1* expression, which may be a mechanism to combat increased inflammation within the microenvironment (179) while also potentially enhancing the self-renewal and survival of pre-malignant clones. The precise mechanisms whereby macrophages regulate marrow inflammation and regeneration will likely provide insight to SAA and MDS pathophysiology.

## Macrophages Regulate HSC Dysfunction in Aging

Normal aging is associated with a spectrum of disorders and increased frailty that correlates with a general escalation of systemic inflammation. Dysfunction of the hematopoietic system in aged individuals includes diminished immune responses and weakened host defense, altered lineage output, anemia, clonal hematopoiesis, and increased incidence of myeloid neoplasms all of which are due to combinatorial intrinsic and extrinsic mechanisms [reviewed in (180, 181)]. In aged animals HSCs exhibit decreased self-renewal, enhanced myeloid and megakaryocytic differentiation, and have reduced homing abilities (182, 183). Dramatic remodeling occurs in the bone marrow of aged animals with an increase in endothelial volume, increased neurovascularization, and reduced collagen, correlating with age-associated bone loss (184, 185). HSCs have been shown to relocate to perisinusoidal niches, where they become less quiescent and adopt myeloid/megakaryocyte bias (184, 186). In many ways, the low level of inflammation associated with aging recapitulates observations of hematopoietic output seen in acute inflammation, namely reduced lymphopoiesis and enhanced myelopoiesis.

The incidence of bone marrow failure, myeloid cancers, and clonal hematopoiesis increases with age, which suggests a direct relationship between elevated inflammatory signaling and disease progression. Increasing age is also associated with clinical challenges as seen in aged individuals with SAA, where effectiveness of immunosuppressive therapies is greatly diminished and the only curative treatment, HSC transplantation, is associated with increased risk of complications and failure (187). Macrophages are sources of inflammatory molecules and also respond to specific inflammatory factors, such as G-CSF (109), suggesting their potential to influence HSC function and hematopoietic output during aging.

Macrophage dysfunction in aging plays a prominent role in cardiovascular disease, cancer, and autoimmune diseases (188). In the bone marrow, macrophage dysfunction induced platelet-biased HSCs in aged mice (185). Macrophages from aged marrow exhibited a pro-inflammatory transcriptional signature, whereas genes associated with phagocytosis and efferocytosis were markedly reduced (185). The ability of marrow macrophages to clear senescent neutrophils was impaired in aged mice, and reduced phagocytosis corresponded to elevated transcription of pro-inflammatory genes, including *Il1b*. The phagocytic program observed in aging was consistent with studies in young mice that demonstrated cell clearance dampens inflammation and imprints a program of continued clearance (189), thus supporting a direct relationship between increased inflammation and reduced phagocytosis in aging. Moreover, deficiency in Axl recapitulated the age-associated phenotypes. IL-1R signaling is a key driver of inflammation-induced HSC dysfunction (9), and aged marrow macrophages not only expressed elevated *Il1b* transcripts but also contained active caspase 1, necessary for processing active IL-1β. It will be important to determine if the impact of IL-1R signaling on platelet-bias is an HSC-intrinsic mechanism, or if autocrine or paracrine signaling in other cell types is critical for HSC dysfunction and, furthermore, how IL-1R signaling in distinct marrow niches impacts HSC biology. As inflammation can impair efferocytosis and efferocytic activity can dampen inflammation, this circuitry may be a critical target for diminishing inflammation in aging.

Inflammation is a critical driver of overt hematologic dysfunction and is emerging as an underlying driver of clonal expansion in the hematopoietic compartment, and likely other tissues. Clonal hematopoiesis, while not necessarily sufficient to induce hematologic disease, has been demonstrated to impact mortality associated with cardiovascular disease (190). Therefore, understanding the intrinsic and extrinsic cues regulating clonal evolution within the hematopoietic system is broadly relevant to human health. Tet2 loss of function is an important driver of clonal hematopoiesis and studies in mice have demonstrated that Tet2-deficient macrophages accelerated cardiovascular disease (191). Clonal hematopoiesis is interrogated by examining the peripheral blood where variant allele frequencies are most common among myeloid cells. The clonal nature of the marrow microenvironment is an important consideration in understanding how selection and outgrowth occurs, as mature myeloid cells may feedback on the more primitive progenitors through production of inflammatory factors.

## Summary and Open Questions

Hematopoiesis is a dynamic process that adapts rapidly to inflammatory stress in order to provide cells that are in high demand. The flexibility of this elegant system is necessary for host defense and responses to trauma and injury. The system also has built-in mechanisms to restore homeostasis, though these are not well-understood. Macrophages contribute to the regulation of HSC biology during all stages of life, and it is now established that macrophages control hematopoietic output in health and disease and are critical regulators in diverse pathologic states. However, key questions remain about bone marrow macrophages. Are there inherent differences between embryonically derived and HSC-derived bone marrow macrophages? How are marrow macrophages maintained or replaced in health and in response to inflammation and infection? How does location in the bone marrow impact macrophage function? What are the key signals that promote pathologic macrophage survival? Are macrophages that derive from specific mutant HSC clones more able to self-renew *in vivo*? Can HSC transplantation be improved via targeting marrow macrophages in some way? Exploring these and other gaps in our understanding may reveal useful insights to our fundamental understanding of HSC biology, but also provide windows of opportunity to reverse disease and maintain healthy macrophages and hematopoiesis throughout life.

## Author Contributions

AS, JM, and KM reviewed literature and wrote the manuscript. All authors contributed to the article and approved the submitted version.

## Conflict of Interest

The authors declare that the research was conducted in the absence of any commercial or financial relationships that could be construed as a potential conflict of interest.
